# Guiding the failing heart to exercise

**DOI:** 10.1007/s12471-014-0637-6

**Published:** 2014-12-05

**Authors:** S. Gielen, D. Merkus, D. J. Duncker

**Affiliations:** 1 Department of Internal Medicine III, Martin-Luther-University Halle/Wittenberg, University Hospital, Halle/Saale, Germany; 2 Division of Experimental Cardiology, Department of Cardiology, Thoraxcenter, Cardiovascular Research Institute COEUR, Erasmus MC, University Medical Center Rotterdam, PO Box 2040, 3000 CA Rotterdam, the Netherlands

The benefit of exercise training in congestive heart failure (CHF) has become widely appreciated over the past 20 years. It is now clear that exercise exerts a number of beneficial effects in CHF, including an improvement of skeletal muscle perfusion, and metabolism, breathing efficiency, and neurohumoral activation [1]. More recent evidence indicates that exercise training in patients with CHF also improves cardiac function [2], which is in agreement with observations in animal models of CHF and appears mediated by exercise-induced amelioration of CHF-associated interstitial fibrosis and cardiomyocyte dysfunction and apoptosis [3]. These beneficial effects of exercise are the consequence of the activation of different molecular signalling pathways that drive the transcriptional control of cardiac remodelling in exercise training versus CHF [1, 3, 4].

In this issue of the Netherlands Heart Journal the *Dutch Royal Society for Physiotherapy* (KNGF) presents practical guidelines for exercise-based cardiac rehabilitation of patients with CHF [5]. Interestingly, the number of national guidelines in clinical cardiology in most European countries is currently declining, which is due to a number of reasons. First, scientific collaboration among national cardiology societies in Europe has become very close within the European Society of Cardiology (ESC) and even large countries such as Germany now no longer produce national guidelines, e.g. for myocardial revascularisation, but issue a German translation of the ESC pocket guideline accompanied by a ‘commentary’ that puts ESC recommendations into a national perspective. Second, the scientific standards to produce guidelines have been continually raised over the last two decades: To assess the evidence base a large and continually increasing number of published studies need to be assessed for quality in order to be included in meta-analyses. This process can only be accomplished with professional bibliographic and statistical help and requires significant resources. So why did the *Dutch Royal Society for Physiotherapy* engage in the painstaking task of producing national guidelines for exercise-based cardiac rehabilitation (CR) of patients with chronic CHF? In contrast to most areas of acute care, the financing, infrastructure, and medical quality of the CR program differ significantly between European countries. It may therefore make sense to develop a practical guideline particularly targeted to the clinical situation in the Netherlands. Additionally, the *Dutch Royal Society for Physiotherapy* represents physiotherapists rather than clinical cardiologists and therefore takes a different perspective of allied health personnel on how to implement the treatment strategies that cardiologists or rehabilitation physicians may have recommended. Consequently, it is much more detailed than usual cardiology guidelines in the practical aspects of patient functional assessment, patient education and empowerment, and the actual training program [5, 6].

The key messages of these guidelines are: (*i*) Aerobic endurance training at 50–80 % of VO_2_ peak remains the training intervention of choice for most CHF patients, especially those in advanced stages of functional impairment. High-intensity interval training (HIT) can be recommended in relatively low-risk CHF patients to achieve higher training effectiveness. (*ii*) Objective assessment of maximal functional capacity by cardiopulmonary exercise testing is a precondition for optimal individual determination of recommended training intensities. (*iii*) Useful optional components of the training programme may comprise inspiratory muscle training, strength training, and relaxation therapy. Patient assessment can help to identify patients who benefit from these program components (i.e. Pimax < 70 % predicted, significant muscle wasting, high anxiety levels). (*iv*) Exercise training is only effective as long as it is maintained. Therefore, continuation of regular physical activity and training after the cardiac rehabilitation phase needs to be encouraged and - if possible - monitored.

However, because this is a guideline for physiotherapists it does not address several important issues, which are relevant for the general clinical cardiologist, including: (*i*) How can we identify CHF patients who may particularly benefit from training therapy? (*ii*) How to assess the patient’s individual risk before the initiation of training therapy? (*iii*) What are the inclusion and exclusion criteria for training interventions in CHF? (*iv*) What invasive/non-invasive diagnostic testing should be performed prior to transferral for training therapy? For all these questions, physicians should consult the position paper from the *European Association of Cardiovascular Prevention and Rehabilitation* [7]. In the setting of chronic CHF, peak exercise capacity should be determined by maximal symptom-limited cardiopulmonary exercise testing with small 5 to 10 W/min increments. In known ischaemic heart disease exercise-induced myocardial ischaemia should be determined and exercise levels should be below the ischaemic threshold. If cardiopulmonary exercise-testing equipment is not available, submaximal tests, such as the 6-min walk test, should be considered [8]. A baseline echocardiogram is recommended to assess left ventricular systolic function, and to exclude potentially correctable conditions such as high-grade mitral regurgitation prior to the initiation of training therapy.

Notwithstanding the medical conditions required to qualify for training therapy, it is essential for physicians not to withhold the benefits of rehabilitation from their patients. Thus, current referral rates for cardiac rehabilitation range between 12 % in US Medicare patients and 70 % in middle European countries with well-developed rehabilitation systems [9, 10]. In large reviews on referral strategies and enrolment rates in cardiac rehabilitation, automatic referral orders and liaison methods have been identified as the most useful strategies to improve referral rates [11]. In the end, however, it comes down to the patient’s and the physician’s conviction that cardiac rehabilitation is needed to achieve optimal clinical outcome [12]. Hence, it is up to us to educate all patients with CHF that there is a clear dose–response relation between training intensity (as measured by MET-hours per week) and outcome improvement indicating significant reductions in adjusted hazard ratios for all cause/cardiac mortality or all-cause/cardiac hospitalisation between 3 and 7 MET-hours per week [13].
